# Where are we now?

**DOI:** 10.2807/1560-7917.ES.2024.29.2.2401111

**Published:** 2024-01-11

**Authors:** Eurosurveillance editorial team

**Affiliations:** 1 European Centre for Disease Prevention and Control (ECDC), Stockholm, Sweden

**Keywords:** public health

Traditionally, at the beginning of a new year, we reflect on selected events relevant for public health and related to the journal together with some anticipated future development.

In May 2023, more than 40 months since the emergence of SARS-CoV-2 in late December 2019, the COVID-19 pandemic was officially declared over as global health emergency on 5 May 2023 [1]. Still, COVID-19 has kept scientists, clinicians, public health practitioners and policymakers as well as us editors busy throughout the rest of the year. Indeed, searching for COVID-19 yields 87,219 articles added to PubMed in 2023, *Eurosurveillance* received its 3,000th submission on COVID-19 in October 2023, and the share of submitted articles on COVID-19 to the journal has remained high. Despite the vast number of already published COVID-19 articles, there are still important lessons and new developments. One example is the emergence of a new SARS-CoV-2 variant that has recently become dominant in several countries. In this week’s issue a rapid communication assesses whether the increased spread of the recently emerged SARS-CoV-2 variant JN.1 compared with the BA.2.86 variant, is related to its ability to escape pre-existing immunity. The authors conclude that the documented immunity escape may not be the cause of the recent upsurge in JN.1 incidence and other reasons should be investigated [2].

Public health measures to mitigate the mortality and morbidity of COVID-19 have impacted not only the spread of other respiratory pathogens but also other aspects of infectious disease epidemiology. Among the many related submissions received in 2023, we aimed to select for publication those that we felt brought new insight or that could be adapted to another setting [3-5]. As in previous years, topics covered regularly in the journal included (but were not limited to) results from vaccine effectiveness studies for vaccines against influenza and COVID-19, epidemiology of vaccine-preventable diseases, surveillance of antimicrobial-resistant pathogens, outbreaks of hospital-acquired infections, emergence of vector-borne diseases as well as tuberculosis, HIV, and food- and waterborne diseases.

In 2023, we received 760 submissions and published 139 regular articles, 57 rapid communications and 18 editorials, letters to the editor and meeting reports. Anecdotal feedback indicates that experts value the rapid communications as a well-established channel to alert both public health experts and clinicians to emerging public health matters.

Examples of topics that received special attention in 2023 were the spillover of highly pathogenic avian influenza A(H5N1) virus infections to mammalian species including minks, raccoon dogs, foxes and cats in several countries across Europe [6-9] and botulism outbreaks with unusual features. One rapid communication described in detail 34 patients with iatrogenic botulism following treatment with botulinum neurotoxin for weight reduction in an outbreak comprising at least 87 cases from five countries [10], while another article reported on an international food-borne botulism outbreak linked to an international sports event in France, which involved 15 cases from seven countries including one fatal case [11]. In both outbreaks, investigators were able to overcome challenges in disease detection and demonstrated that international alerts and collaboration were key to identifying and alerting cases.

Easing the lives of authors and editors, spell checkers and similar tools have been used on daily basis for several years; the year 2023, however, brought the breakthrough and wide use of generative artificial intelligence (AI) tools such as chatbots, large language models and image-generating algorithms. These tools are viewed by some editors as a blessing and by others as a curse and, while they present impressive new opportunities, they also come with numerous challenges related to originality, authorship concepts and more generally, publication ethics and responsibility when writing scientific reports. Acknowledging these challenges and taking guidance from editorial standard-setting bodies such as the International Committee of Medical Journal Editors (ICMJE) and the World Association of Medical Editors (WAME), we published a policy on Responsible use of artificial intelligence (AI) tools for our authors and reviewers. We are aware that this field is rapidly evolving, and we will need to be vigilant to learn and to adapt our policies and practices when and where necessary.

Just at the beginning of the summer holiday period, we received good news: the metrics for the journal continued to be favourable. As the highest ranked open access journal with a Journal Impact Factor of 19 for the year 2022, *Eurosurveillance* remained at rank five among the 96 journals listed in the Clarivate Journal Citations Report (JCR) category “Infectious Diseases”, and the SCImago Journal Rank is 44 among 2,499 journals in the category “Medicine (miscellaneous)”. The Scopus-based CiteScore for 2022 went up from 22.1 in 2021 to 27.7, and *Eurosurveillance* is in the 99th percentile of journals in the category “Public Health, Environmental and Occupational Health” where it ranks at the third place among 562 journals. Moreover, a stakeholder survey conducted by our publisher – the European Centre for Disease Prevention and Control (ECDC) – in late 2022, confirmed that quality and reliability were viewed as good or very good by 99% and 100% of respondents, respectively, and 93% deemed that we achieved our aim to provide authoritative public health science to inform policy measures and public health action to “a great extent” (58%) or “some extent” (35%). Stakeholders used our articles for a wide range of purposes including general knowledge, policy and decision making, public health interventions, teaching, research, and clinical practice.

In May 2023, the European Council issued a Conclusion on high-quality, transparent, open, trustworthy and equitable scholarly publishing [12]. As a diamond open access journal, published by a non-for-profit publisher whose director grants the editor-in-chief and the journal editorial independence, *Eurosurveillance* has contributed to early and open dissemination of scientific findings since 1996 and is already compliant with many points listed in the Conclusion. For example, rapid communications, online publishing under a Creative Commons (CC) BY 4.0 licence, and availability in open repositories such as PubMedCentral have long been components of the journal’s policies, enabling the sharing and reuse of scientific data and results in a timely and non-restricted manner. In addition, we do not charge article fees (commonly referred to as article processing charges, APC) to our authors, which has also provided equitable access for authors from countries in Europe where funding for publications may be limited. Moreover, the journal has endorsed important initiatives contributing to transparency and improved quality of science such as the Sex and Gender Equity in Research (SAGER) guidelines and encouraged their use among authors and reviewers [13].

*Eurosurveillance’*s anecdotal and measured success and impact result from a continued joint team effort with our board members, our contributors and assistance from our publisher, as well as with our many allies within and outside the ECDC. We do not take your support for granted and we hope you will continue to act as our advisors and professional ‘friends’ who provide us with guidance and constructive feedback. You help us shape the journal and publish public health-relevant articles of good quality. Our double-anonymised peer review prevents us from acknowledging the valuable expertise and guidance of our peer reviewers at article level. Instead, we published a list with their names for the whole year in alphabetical order in our first 2024 issue to thank and express our gratitude to the 462 experts who dedicated their time to peer-review our articles in 2023 [14].

In 2024, we will continue working with authors, peer reviewers, our board and colleagues and other supporters to select and publish relevant and attractive articles for our audience as evidence for public health action and policymaking, and for research and clinical practice. Through our annual theme *’Changing urban environments and effects on infectious diseases and their epidemiology, surveillance, prevention and control’*, we intend to showcase interdisciplinary experiences, innovative approaches and aspects concerning infrastructural changes to make cities resilient and reach the sustainable development goals (SDGs) by 2030 [15] with focus on how such changes may impact on infectious disease epidemiology. We look forward to receiving many submissions that fit this scope.

Following the earlier pandemic years with limited travel and face-to-face meetings, we enjoyed opportunities to (re-)establish direct contact with our contributors and readers through physical presence at conferences and meetings in 2023. We also reinforced our interactions with our audience through online webinars and training activities and we will continue these activities in 2024. They bring us closer to our audience and enable us to gain new insights through capturing different perspectives and needs. To further increase quality, relevance and impact, we will strengthen aspects of diversity and inclusion in our policies and published articles. During the first months of 2024, we plan to launch online microlearning modules to support peer reviewers for example in assessing diversity and inclusion aspects of a manuscript.

From where we are now and looking into the future, we trust we will collaborate with many contributors from different disciplines and backgrounds on interesting and timely articles of high quality that support public health policy and action.
